# Cost-effectiveness analysis of quadrivalent seasonal influenza vaccines in England

**DOI:** 10.1186/s12916-017-0932-3

**Published:** 2017-09-08

**Authors:** Dominic Thorrington, Edwin van Leeuwen, Mary Ramsay, Richard Pebody, Marc Baguelin

**Affiliations:** 1 Respiratory Diseases Department, Public Health England, 61 Colindale Avenue, London, NW9 5EQ UK; 2 Imperial College Faculty of Medicine, London, SW7 2AZ UK; 3 Immunisation, Hepatitis & Blood Safety Department, Public Health England, 61 Colindale Avenue, London, NW9 5EQ UK; 4 Department of Infectious Disease Epidemiology, London School of Hygiene and Tropical Medicine, Keppel Street, London, WC1E 7HT UK

**Keywords:** Influenza, Vaccination, Quadrivalent vaccines, Cost-effectiveness, QALY, LAIV

## Abstract

**Background:**

As part of the national seasonal influenza vaccination programme in England and Wales, children receive a quadrivalent vaccine offering protection against two influenza A strains and two influenza B strains. Healthy children receive a quadrivalent live attenuated influenza vaccine (QLAIV), whilst children with contraindications receive the quadrivalent inactivated influenza vaccine (QIIV). Individuals aged younger than 65 years in the clinical risk populations and elderly individuals aged 65+ years receive either a trivalent inactivated influenza vaccine (TIIV) offering protection from two A strains and one B strain or the QIIV at the choice of their general practitioner.

The cost-effectiveness of quadrivalent vaccine programmes is an open question. The original analysis that supported the paediatric programme only considered a trivalent live attenuated vaccine (LAIV). The cost-effectiveness of the QIIV to other patients has not been established. We sought to estimate the cost-effectiveness of these programmes, establishing a maximum incremental total cost per dose of quadrivalent vaccines over trivalent vaccines.

**Methods:**

We used the same mathematical model as the analysis that recommended the introduction of the paediatric influenza vaccination programme. The incremental cost of the quadrivalent vaccine is the additional cost over that of the existing trivalent vaccine currently in use.

**Results:**

Introducing quadrivalent vaccines can be cost-effective for all targeted groups. However, the cost-effectiveness of the programme is dependent on the choice of target cohort and the cost of the vaccines: the paediatric programme is cost-effective with an increased cost of £6.36 per dose, though an extension to clinical risk individuals younger than 65 years old and further to all elderly individuals means the maximum incremental cost is £1.84 and £0.20 per dose respectively.

**Conclusions:**

Quadrivalent influenza vaccines will bring substantial health benefits, as they are cost-effective in particular target groups.

**Electronic supplementary material:**

The online version of this article (doi:10.1186/s12916-017-0932-3) contains supplementary material, which is available to authorized users.

## Background

Seasonal influenza is a major public health problem in England. It is estimated that approximately 10% of all respiratory hospital admissions and deaths can be attributed to circulating influenza, with the highest admission rates for both influenza A and B strains attributed to children under 5 years of age and the highest influenza-attributed deaths rates seen in the group of elderly patients with co-morbidities [1].

Two antigenically and genetically distinct influenza B lineages emerged in the early 1980s and have since spread globally, co-circulating every influenza season [2]. An analysis published in 2014 reported that children younger than 15 years bear the largest burden of disease due to influenza B strains in England, with 1744 annual general practitioner (GP) consultations per 100,000 (95% confidence interval, CI 1656–1832) for those children aged 5–14 years [1]. However, the burden of GP consultations attributed to influenza B infection for older members of the population is not insignificant, with 552 (95% CI 528–576) and 361 (95% CI 340–382) GP consultations per 100,000 per year for individuals aged 15–44 years and 45–65 years respectively. However, no GP consultations or hospitalisations for those individuals aged 65 years and older were attributed to influenza B on average each season.

In England, seasonal influenza vaccination is offered to several population groups: elderly individuals aged 65 years and older; clinical risk groups; and more recently the Department of Health has started the incremental introduction of the offer of vaccination to healthy children aged 2–16 years. A cost-effectiveness analysis [3] demonstrated that a trivalent live attenuated influenza vaccine (LAIV) offered to this cohort would be very cost-effective (£1949 per quality-adjusted life year, QALY) and would bring substantial public health benefits in the form of reduced health care resource use through both direct protection to children and indirect protection to both the clinical risk groups and elderly individuals. The programme is not being rolled out to all children aged 2–16 years at once but to incremental age bands each year in England, with all children aged 2–7 years offered the vaccine in 2016–2017 [4] with all children up to age 11 years to be offered vaccination soon. In 2014–2015 the programme was implemented with a quadrivalent live attenuated influenza vaccine (QLAIV) for children without contraindications [5, 6], offering protection against two influenza A strains and two influenza B strains. For all individuals in clinical risk groups the Joint Committee on Vaccination and Immunisation (JCVI) recommends the quadrivalent inactivated influenza vaccines (QIIVs) as being preferable to the trivalent inactivated influenza vaccines (TIIVs) with all other things being equal [7].

GPs and pharmacists are able to procure QIIVs from various manufacturers for their adult patients in clinical risk groups, defined as those with chronic respiratory, heart, kidney, liver or neurological disease, diabetes, immunosuppression or asplenia [7] with morbid obesity added to the list from 2017–2018 [8, 9]. The vaccines are purchased from manufacturers directly before GPs and pharmacists are reimbursed. Quadrivalent vaccines administered to those individuals in clinical risk groups and to the elderly population may reduce influenza B-attributed morbidity and mortality, as recent randomised controlled trials (RCTs) have demonstrated the superiority of quadrivalent vaccines over trivalent vaccines in both adults [10] and children [11]. The cost-effectiveness of dispensing quadrivalent influenza vaccines to clinical risk groups and the elderly population should be assessed in light of the successful paediatric seasonal influenza vaccination programme that will soon include all children attending primary schools.

This analysis considers the direct and indirect impact of administering an inactivated quadrivalent seasonal influenza vaccine to those individuals aged < 65 years in clinical at-risk groups and subsequently to all elderly individuals, in addition to assessing the impact of the paediatric programme administering QLAIVs to healthy children and QIIVs to those children with contraindications compared to the predicted impact of the trivalent vaccines. We sought to establish the maximum incremental cost per dose of the quadrivalent vaccines over existing trivalent vaccines to all targeted cohorts.

## Methods

### Modelling approach

We used the same mathematical model as the one in [3] and [12], used to recommend the introduction of the paediatric seasonal influenza vaccination programme in the UK. The model brings together surveillance data from a variety of primary care, secondary care and sentinel data sources in addition to data on social contact patterns and seroepidemiological data and uses these data in a Bayesian approach, specifically adaptive Markov chain Monte Carlo (MCMC) techniques, to reconstruct epidemics using a transmission model for three influenza subtypes over 14 years. The model simulated seasonal influenza outbreaks in seven age groups (<1 year, 1–4, 5–14, 15–24, 25–44, 45–64 and 65+ years) and two at-risk groups (low risk, clinical risk). All parameters for both the transmission model and economic evaluation model remained the same from both publications. We assumed that the paediatric programme had been implemented for all primary schools only and simulated epidemics using the model through the fluEvidenceSynthesis R package [13].

The model follows a modified Susceptible-Exposed-Infectious-Removed (SEIR)-type structure with gamma-distributed latent and infectious periods. At the beginning of an influenza outbreak, a small fraction of individuals in each age class is infectious with the remainder susceptible to infection. Mixing between clinical risk groups is random within each age group, with the population mixing patterns described via a resampling of the POLYMOD matrix for the population of Great Britain [14]. Costs and health benefits from the perspective of the health care provider were discounted an annual rate of 3.5%. The full model and cost-effectiveness framework are described in greater detail in both [3] and [12].

**Fig. 1:**
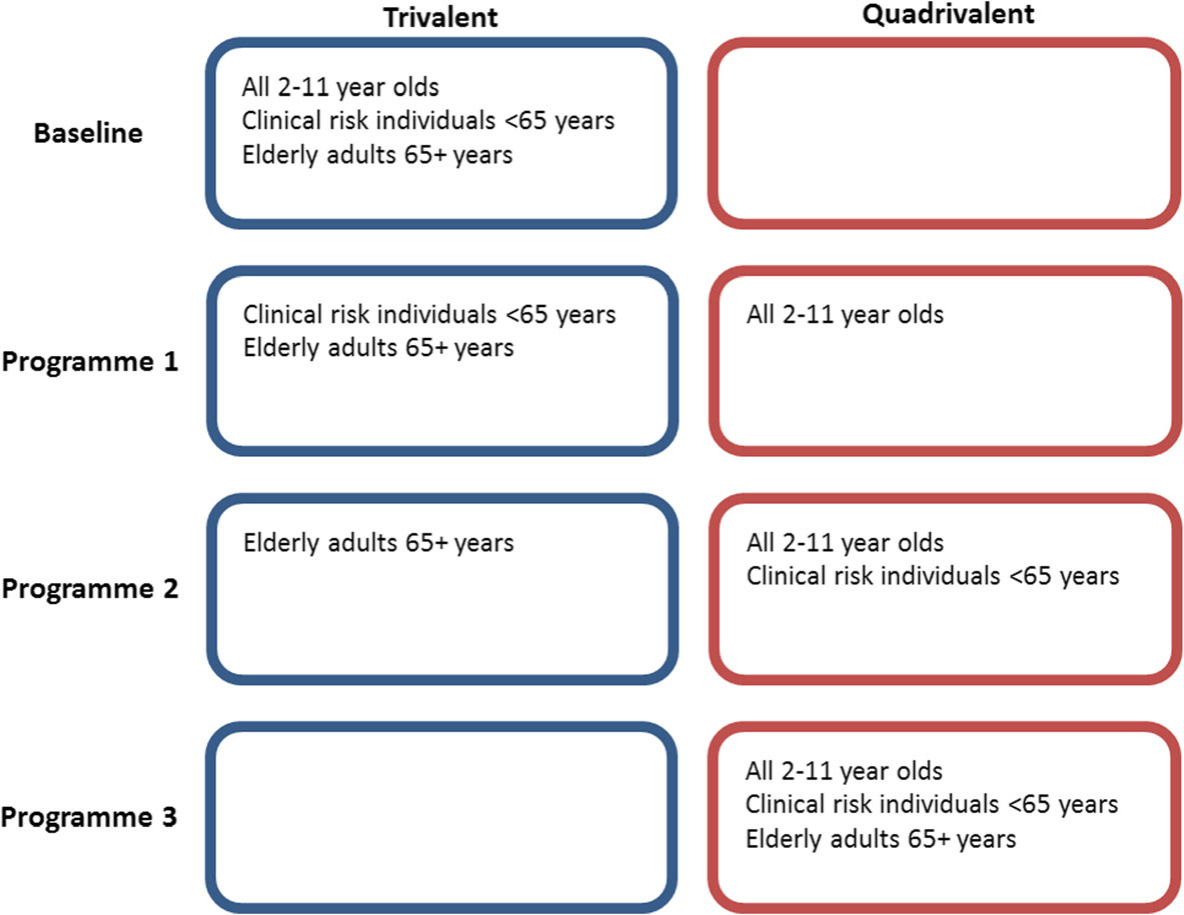
Scenarios simulated in the model

We simulated four scenarios in the model, described below and shown in Fig. 1.

#### Baseline

The baseline scenario is as follows:

- Low-risk young and primary school children (aged 2–11 years) receive the trivalent LAIV, which offers protection from two influenza A strains and one influenza B strain.
- Clinical risk and elderly individuals receive the inactivated trivalent vaccine, offering protection from two influenza A strains and one influenza B strain. This simulates the initial cost-effectiveness analysis described in [3], though with the paediatric vaccination programme rolled out to only the primary schools. All healthy children are eligible for the seasonal influenza vaccine and they receive the trivalent LAIV, whilst clinical risk and elderly individuals continue to receive the inactivated trivalent vaccine.

#### Programme 1

Under programme 1, low-risk children without contraindications aged 2–11 years receive the QLAIV, which offers protection from two influenza A strains and two influenza B strains. Children with contraindications receive the QIIV. This modelling scenario depicts the current seasonal influenza vaccination programme.

#### Programme 2

Under programme 2, low-risk children aged 2–11 years and the clinical risk individuals aged < 65 years receive a quadrivalent vaccine (QLAIV for low-risk children, QIIV for contraindicated children and clinical risk individuals < 65 years). This modification to the current programme sees protection against an additional strain offered to clinical risk individuals aged < 65 years, with the additional cost of the QIIV paid in reimbursements to GPs and pharmacists who administer the vaccine to persons < 65 years old in their surgeries.

#### Programme 3

Under programme 3, low-risk children aged 2–11 years and clinical risk individuals of all ages in addition to all individuals aged 65 years and older receive a quadrivalent vaccine, ensuring that all eligible individuals in England are offered a quadrivalent vaccine (QLAIV for low-risk children without contraindications and QIIV for all other eligible individuals).

Finally, we simulated all four scenarios again with the addition of a paediatric vaccination programme that had been fully rolled out to include all children aged 2–16 years.

### Model parameters

No efficacy studies have been published on the QIIV [15]. The efficacy of the QLAIV used in low-risk children has not yet been demonstrated in clinical trials. The manufacturers of the inactivated vaccine also manufacture two trivalent influenza vaccines formulated with the same two influenza A strains (both A/H1N1 and A/H3N2) but different B strains (Victoria lineage and Yamagata lineage respectively). The efficacies of these vaccines are considered to be good predictors of the efficacy of the QIIV, as the manufacturing process for all strains was unchanged from that of the trivalent vaccines [16]. We therefore assumed equivalent efficacy for the QIIV, where the trivalent vaccine was poorly matched to the circulating influenza B strains and the quadrivalent vaccine was well matched, using the same parameters from [12] and [3] listed in Table 1.

**Table 1:** Parameters used in the dynamic transmission model

Parameter	Value and uncertainty	Source
Efficacy against B strain(s)		
TIIV and LAIV, < 65 years	42%	[3]
TIIV and LAIV, 65+ years	28%	[3]
QIIV and QLAIV, < 65 years §	70% (min. 50%, max. 90%)	[3]
QIIV and QLAIV, 65+ years §	46% (min. 30%, max. 70%)	[3]
Influenza vaccination coverage		
Low-risk < 5 years §	33.7% (±10%)	[17]
Low-risk 5–16 years §	54.9% (±10%)	[17]
Clinical risk < 6 months–64 years §	45.1% (±10%)	[17]
65+ years §	71.0% (±10%)	[17]
Health care resource costs		
GP consultation	£46, lognormal (mean 46, standard deviation 8.4)	[3]
Inpatient admission	£1050, lognormal (mean 1050, standard deviation 192.1)	[3]

The § symbol denotes that the parameter was used in the sensitivity analysis

Vaccination coverage data for all age and risk groups were taken from the monthly coverage data published by Public Health England [17]. The data for the final coverage achieved are shown in Table 1. The primary and tertiary care costs from [3] were updated.

We assumed that the new quadrivalent vaccine administered to the clinical risk groups and elderly individuals would cost the same per dose as the quadrivalent vaccine administered to school children. The maximum incremental cost per dose of the quadrivalent vaccines over the trivalent vaccines was estimated using a threshold analysis on the mean incremental cost-effectiveness ratios for each proposed vaccination programme for a series of potential willingness-to-pay (WTP) thresholds. The incremental vaccine costs were varied from no difference to £15. The criteria to be cost-effective were in line with recommendations from the JCVI that 90% of simulations had an incremental cost-effectiveness ratio below the WTP threshold [18].

Epidemics for each vaccination programme were simulated 1000 times. We estimated the mean savings in primary care and tertiary care, reporting 95% CIs of the means based on 1000 bootstrap replications.

### Sensitivity analysis

We conducted a sensitivity analysis using the highlighted parameters (indicated with §) in Table 1 to assess the sensitivity of our estimates to parameter uncertainty. The efficacy of the quadrivalent vaccines in preventing infection from influenza B is unclear, so we assumed a variation of ±20% in absolute terms. The uptake for all three target cohorts was varied by ±10% in absolute terms. In addition, we varied the proportion of infections that attend primary care consultations with influenza-like illness (ILI) symptoms by ±50% of that reported in [1]. The impact of these uncertainties was assessed against the most cost-effective incremental cost of the quadrivalent vaccines with a WTP threshold of £20,000 per QALY.

## Results

**Fig. 2:**
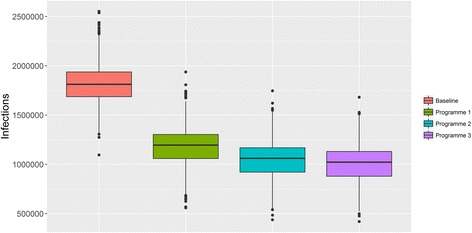
Distribution of the number of infections for each vaccination scenario

Figure 2 shows the variation in the distribution of ILI cases across the modelled scenarios. Using the trivalent LAIV for low-risk school children as well as a trivalent inactivated vaccine in all other eligible groups resulted in approximately 1,819,711 infections (95% CI 1,807,163–1,831,307). The introduction of the QLAIV to low-risk school children without contraindications alongside the QIIV for those children with contraindications reduced the case burden caused by ILI infection by 35% to 1,176,059 (95% CI 1,163,850–1,189,061). Simulating the introduction of the quadrivalent inactivated vaccine to clinical risk individuals under 65 years old reduced the case burden to 1,039,774 (95% CI 1,028,029–1,051,566), and the addition of those individuals aged 65 years and older further reduced the case burden to 1,000,477 (95% CI 989,314–1,011,567). A full breakdown of the estimated incremental benefits of each proposed vaccination programme is presented in Table 2.

**Table 2:** Mean incremental benefits of each proposed vaccination programme

Parameter	Mean reduction after starting programme 1 (standard deviation)	Mean reduction after starting programme 2 (standard deviation)	Mean reduction after starting programme 3 (standard deviation)
Infections	643,652 (110,710)	136,284 (21,246)	39,297 (16,105)
Symptomatic/febrile cases	59,457 (20,816)	12,578 (4264)	3641 (1942)
GP consultations	63,276 (10,680)	11,305 (1757)	2658 (1123)
Hospitalisations	356 (64)	49 (9)	14 (6)

If the WTP threshold is £20,000 per QALY, then the maximum incremental cost of the quadrivalent vaccine for school children is £6.36. Further extension to clinical risk individuals younger than 65 years old and to all individuals aged 65+ years resulted in maximum incremental costs of £1.84 and £0.20 respectively. Increasing the WTP threshold to £30,000 per QALY means that each of the three proposed vaccination programmes would be cost-effective with maximum incremental costs of £8.89, £2.66 and £0.31 respectively.

The maximum incremental cost of the quadrivalent vaccines that ensures a cost-effective programme for all targeted cohorts is very small because of the projected health care resource use of the elderly population infected with influenza B. Extending a quadrivalent vaccination programme to all clinical risk and elderly individuals reduces the number of febrile influenza B cases by 16,218 with projected savings in primary and secondary care, but at the increased cost of vaccinating nearly 9,000,000 individuals of whom 74% are aged 65 years or older and less likely to require primary or secondary care intervention.

### Additional results assuming all children aged 2–16 years are eligible

If the paediatric vaccination programme is extended to all children of ages 2–16 years, then the maximum incremental costs of the quadrivalent vaccines in each proposed programme would be £4.04, £1.03 and £0.06 respectively.

### Model sensitivity

**Fig. 3:**
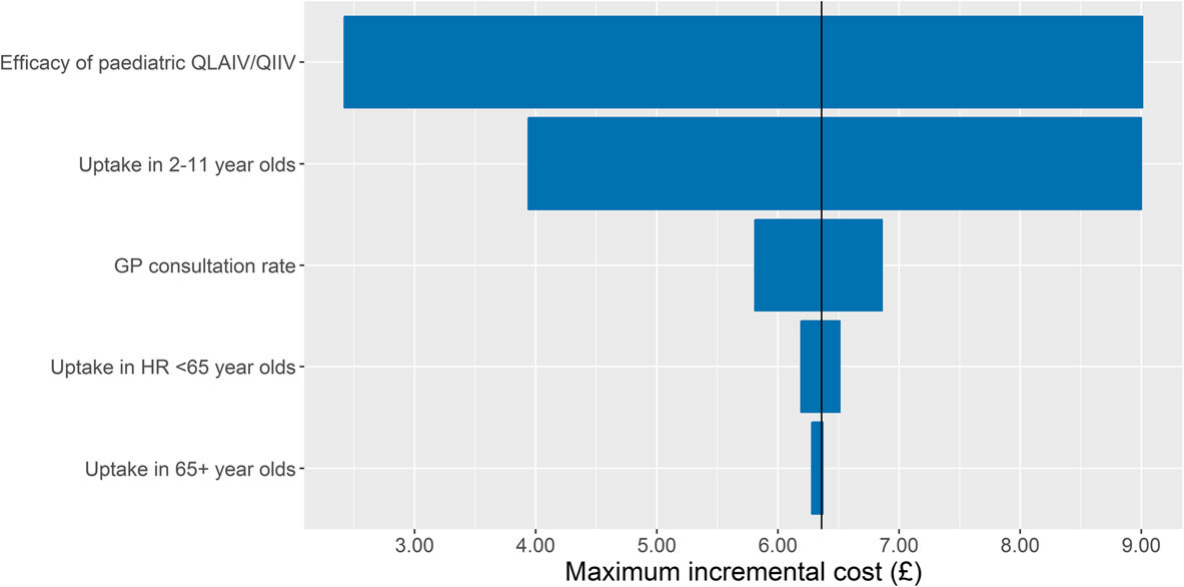
Tornado plot showing the estimated change in the maximum incremental cost per dose of the quadrivalent vaccines for programme 1, assuming a WTP threshold of £20,000 per QALY

It is clear from the results presented in Table 3 that the programme that allows the largest variation in costs between the trivalent and quadrivalent vaccines is the paediatric programme. The cost-effectiveness of this programme is most sensitive to the efficacy of the paediatric QLAIV/QIIV to the B strains. If the vaccine used for children was more efficacious at preventing influenza B infection, then the maximum incremental cost of the vaccine would be £9.01, whilst a less efficacious vaccine would need to be £2.42 more expensive than the paediatric LAIV/TIIV to remain cost-effective (Fig. 3). Variation in the uptake of the vaccine in this age group by ±10% in absolute terms is also a source of uncertainty for the maximum incremental cost of the QLAIV. In comparison, the rate of GP consultations attributable to ILI had little impact on the cost-effectiveness of the programmes as did the uptake of trivalent vaccines in the older age groups.

**Table 3:** The maximum incremental vaccine cost to ensure that 90% of all simulations are cost-effective to five different WTP thresholds

Cost per QALY	Programme 1	Programme 2	Programme 3
£10,000	£3.69	£1.03	£0.11
£15,000	£5.04	£1.44	£0.16
£20,000	£6.36	£1.84	£0.20
£25,000	£7.58	£2.25	£0.26
£30,000	£8.89	£2.66	£0.31

Other parameters such as the efficacy of the QIIV for the adult age groups cannot have an impact on the cost-effectiveness of the paediatric programme. Their impact on the cost-effectiveness of the adult QIIV programmes is explored in Additional file 1.

## Discussion

We found that the introduction of quadrivalent influenza vaccines to the seasonal influenza vaccination programme in England can be cost-effective for all targeted cohorts, though the cost-effectiveness of the programme implemented is highly dependent on the increased cost of replacing trivalent vaccines with their quadrivalent counterparts. Increasing the eligibility for quadrivalent vaccination programmes further than just the paediatric programme reduces the additional amount that the National Health Service (NHS) should pay to procure each vaccine dose. Indeed, if only trivalent vaccines were available for individuals aged 17 years and younger, then the maximum incremental cost per dose of QIIV vaccines for high-risk and elderly individuals would increase to £3.55 and £0.29 respectively. This increases further to a minimum of £3.76 and £0.41 respectively if no paediatric influenza vaccination programme had been implemented at all, but these latter estimates are likely to be underestimates, as they ignore the substantial change in the population-wide burden of influenza A attributable to the LAIV/TIIV paediatric programme [5, 19].

The maximum incremental cost per dose of the quadrivalent vaccine for high-risk and elderly individuals reduces further from £1.84 and £0.20 if the paediatric influenza vaccination programme is ultimately implemented for all age groups in schools as planned. This is due to two factors: first the heterogeneous burden of influenza B in the population is concentrated in the younger age groups, so a vaccination programme that targets all children of ages 2–16 years will reduce a large proportion of this total burden. Second, the well-demonstrated indirect effect of vaccinating children to protect other age groups reduces the potential impact of any vaccination programme in the remaining population, thereby reducing the amount that should be spent on those vaccination programmes after a successfully implemented paediatric programme. Any additional resources required to implement the paediatric influenza vaccination programme would be the same whether the programme used a trivalent or quadrivalent live attenuated vaccine, so we did not consider this potential additional cost in our analysis.

### Strengths and weaknesses of this study

This study uses a dynamic transmission model that was fitted to 14 years of surveillance data, and it uses the same cost-effectiveness framework that was used to support the decision to implement a nationwide seasonal influenza vaccination programme for healthy children [12], providing a consistent approach to understanding the impact of an evolving vaccination programme. This approach, as outlined in the previous publications, accounts not just for the direct protection inferred on vaccinated individuals but also the indirect protection inferred on unvaccinated individuals in the population. The model also uses the same data [14] on social contacts to account for mixing patterns in the population, an important inclusion to understanding infectious disease dynamics [20].

We assumed that existing trivalent vaccines were poorly matched to the dominant circulating B strain in each influenza season. Whilst this assumption allowed us to maintain a consistent modelling approach with that of Baguelin et al., we recognise that it presents the best-case scenario for introducing quadrivalent vaccines. In addition, other countries have reported that some vaccines were well matched against the dominant circulating influenza B strains with little activity for the unmatched B strain [21, 22], which would reduce the maximum incremental cost per dose of quadrivalent vaccines in our analysis. However, our approach allows us to report the maximum incremental cost in the best possible scenario for quadrivalent vaccines with the understanding that any previous years with a better strain match in vaccines will reduce the cost-effectiveness of quadrivalent vaccines. Indeed, the estimated proportion of influenza B infections between 2000 and 2010 caused by vaccine mismatched strains was 52.4% [23], presenting an encouraging opportunity for quadrivalent vaccines to reduce the public health impact of seasonal influenza.

We did not consider extending eligibility to children aged younger than 2 years old, in contrast to paediatric vaccination programmes in some other countries. The live attenuated vaccine is not licensed for children in this age group; and the effectiveness of inactivated vaccines is lower [24]; and implementation of a programme using an injectable vaccine would represent a considerable additional workload for a policy that has not been recommended in the UK.

Our economic evaluation of the vaccination programmes followed the guidelines recommended by the National Institute for Health and Clinical Excellence (NICE) in evaluating the costs of the programme from the perspective of the health care provider [25]. We took a conservative approach in assessing the potential additional expenditure appropriate to reducing the disease burden caused by influenza B strains. We considered vaccination programmes with at least 90% of all simulations below the WTP threshold as cost-effective, with a WTP threshold of £20,000 per QALY as recommended by NICE. We also reported our results for other WTP thresholds should that advice change in the future.

Our sensitivity analysis reported that our model estimates are most sensitive to the estimated efficacy of the QLAIV in preventing influenza B infection, but the manufacturers of the vaccine have not confirmed these estimates and have instead hypothesised that the similarity in manufacturing processes between the LAIV and QLAIV implies that there is no difference. Greater clarity on the estimated efficacy of the QLAIV in preventing influenza B infection would greatly improve the accuracy of our estimates.

A recent systematic review of economic evaluations assessing the impact of quadrivalent influenza vaccines compared the modelling approaches and results of 16 analyses published before September 2016 [26]. The authors reported that 13 of these analyses were funded by vaccine manufacturers, two did not specify the funding source and the one remaining study was publicly funded. Our independent study is therefore a helpful addition to the literature on the cost-effectiveness of quadrivalent vaccines that is so dominated by industry-funded studies. In addition, the review of de Boer et al. calls for more extensive use of dynamic transmission models to fully understand the impact of quadrivalent vaccines in influenza vaccination programmes, an approach that we adopted here using the fluEvidenceSynthesis R package.

### Relation of this study to other studies

The review of de Boer et al. reported that the range of incremental cost per dose of quadrivalent vaccines over existing trivalent equivalents for all studies was $1.25 to $7.14 in 2015 US dollars [26], though there was variation in the perspective for which economic evaluations were performed, the WTP thresholds for each country and the requirement of the proportion of simulations that should be below those WTP thresholds. Many studies reported that quadrivalent vaccines are cost-effective with an emphasis of the sensitivity of these estimates to the parameters for the cost of vaccines and the efficacy of vaccines considered against the circulating influenza B strains. We adopted a conservative approach to our economic evaluation and feel that our conservative incremental cost-per-dose estimates for each proposed programme compare well to those of the studies featured in this review, with similar findings from our sensitivity analysis to those featured in the review.

### Possible explanations and implications for clinicians and policymakers

Extending a QIIV programme to include clinical risk individuals younger than 65 years old in England is more likely to be cost-effective than extending further to also include all elderly individuals. However, the maximum incremental cost per dose of the quadrivalent vaccines is just £1.84, meaning that the current policy of reimbursing GPs and pharmacists for administering QIIVs to clinical risk groups is likely to be cost-effective if the incremental reimbursement cost is less than this value and less than £0.20 per dose if extended further to all elderly individuals. This result is influenced by the heterogeneous burden of influenza B infection in England [1], with children shouldering the largest proportion of this burden; therefore, directly protecting the elderly from infection with QIIV offers a smaller return than the QLAIV programme in schools.

### Unanswered questions and future research

We did not address the issue of adverse events arising from vaccination using the QIIV and QLAIV and assumed that these risks were negligible, as clinical trials suggest that the quadrivalent vaccines have similar safety profiles to those of the trivalent vaccines [11, 27, 28]. We did not consider the implications of infection from natural exposure to influenza B, nor did we attempt to estimate the impact of repeated vaccination on the immunogenic response in the patient.

## Conclusions

According to our analysis, the introduction of the QLAIV to healthy children is likely to be cost-effective if the quadrivalent vaccine costs no more than £6.36 more than the trivalent live attenuated vaccine that was introduced in 2012. Given the projected reduction in the population-wide burden of influenza B, the introduction of quadrivalent influenza vaccines to the seasonal influenza vaccination programme for clinical risk groups and particularly elderly individuals is likely to be cost-effective only in narrow circumstances. The cost to reimburse GPs and pharmacists administering the QIIV to clinical risk groups needs to be evaluated in light of these findings.

## Additional file

Additional file 1: Assessing the impact of different paediatric programmes, sensitivity analyses for programmes 2 and 3, and cost-effectiveness acceptability curves. (DOCX 44 kb)

## Abbreviations

GP: General practitioner
JCVI: Joint Committee on Vaccination and Immunisation
LAIV: (Trivalent) live attenuated seasonal influenza vaccine
MCMC: Markov chain Monte Carlo
NHS: National Health Service
NICE: National Institute for Health and Clinical Excellence
QALY: Quality-adjusted life year
QIIV: Quadrivalent inactivated (seasonal) influenza vaccine
QLAIV: Quadrivalent live attenuated (seasonal) influenza vaccine
RCT: Randomised controlled trial
SEIR: Susceptible-Exposed-Infectious-Removed compartmental mathematical model
TIIV: Trivalent inactivated (seasonal) influenza vaccine
UK: United Kingdom
WTP: Willingness-to-pay

## References

[1] Cromer D, van Hoek AJ, Jit M, Edmunds WJ, Fleming D, Miller E (2014). The burden of influenza in England by age and clinical risk group: A statistical analysis to inform vaccine policy. J Infect..

[2] Glezen WP, Schmier JK, Kuehn CM, Ryan KJ, Oxford J (2013). The burden of influenza B: a structured literature review. Am J Public Health..

[3] Baguelin M, Camacho A, Flasche S, Edmunds WJ (2015). Extending the elderly- and risk-group programme of vaccination against seasonal influenza in England and Wales: a cost-effectiveness study. BMC Med..

[4] Public Health England. The national flu immunisation programme 2016/17. 2016. https://www.gov.uk/government/uploads/system/uploads/attachment_data/file/529954/Annual_flu_letter_2016_2017.pdf. Accessed 13 Oct 2016.

[5] Pebody R, Warburton F, Andrews N, Ellis J, Von Wissmann B, Robertson C, et al. Effectiveness of seasonal influenza vaccine in preventing laboratory-confirmed influenza in primary care in the United Kingdom: 2014/15 end of season results. Eurosurveillance. 2015;20. doi:10.2807/1560-7917.ES.2015.20.36.30013 25677050

[6] Department of Health, Public Health England, NHS England. Flu plan: winter 2014/15. London, UK; 2014. https://www.gov.uk/government/uploads/system/uploads/attachment_data/file/306638/FluPlan2014_accessible.pdf. Accessed 13 Oct 2016.

[7] Public Health England. Influenza: the green book, chapter 19. 10th ed. 2013. https://www.gov.uk/government/uploads/system/uploads/attachment_data/file/456568/2904394_Green_Book_Chapter_19_v10_0.pdf. Accessed 13 Oct 2016.

[8] JCVI. Minute of meeting on 1 October 2014. 2014. https://app.box.com/s/iddfb4ppwkmtjusir2tc/file/22846051967. Accessed 13 Oct 2016.

[9] Kwong JC, Campitelli MA, Rosella LC (2011). Obesity and respiratory hospitalizations during influenza seasons in Ontario, Canada: a cohort study. Clin Infect Dis..

[10] Kieninger D, Sheldon E, Lin W-Y, Yu C-J, Bayas JM, Gabor JJ (2013). Immunogenicity, reactogenicity and safety of an inactivated quadrivalent influenza vaccine candidate versus inactivated trivalent influenza vaccine: a phase III, randomized trial in adults aged 18+ years. BMC Infect Dis..

[11] Domachowske JB, Pankow-Culot H, Bautista M, Feng Y, Claeys C, Peeters M (2013). A randomized trial of candidate inactivated quadrivalent influenza vaccine versus trivalent influenza vaccines in children aged 3-17 years. J Infect Dis..

[12] Baguelin M, Flasche S, Camacho A, Demiris N, Miller E, Edmunds WJ (2013). Assessing optimal target populations for influenza vaccination programmes: an evidence synthesis and modelling study. PLoS Med..

[13] Leeuwen E van, Klepac P, Thorrington D, Pebody R, Baguelin M. fluEvidenceSynthesis: an R package for evidence synthesis based analysis of epidemiological outbreaks 2016. https://github.com/MJomaba/flu-evidence-synthesis. 10.1371/journal.pcbi.1005838 PMC5714397 29155812

[14] Mossong J, Hens N, Jit M, Beutels P, Auranen K, Mikolajczyk R (2008). Social contacts and mixing patterns relevant to the spread of infectious diseases. PLoS Med..

[15] de Graaf H, Faust SN (2015). Fluarix quadrivalent vaccine for influenza. Expert Rev Vaccines..

[16] Bekkat-Berkani R, Ray R, Jain VK, Chandrasekaran V, Innis BL (2016). Evidence update: GlaxoSmithKline’s inactivated quadrivalent influenza vaccines. Expert Rev Vaccines..

[17] Public Health England. Vaccine uptake guidance and the latest coverage data. London: Public Health England; 2016. https://www.gov.uk/government/collections/vaccine-uptake. Accessed 13 Oct 2016.

[18] JCVI. Code of Practice June 2013. 2013. https://www.gov.uk/government/uploads/system/uploads/attachment_data/file/224864/JCVI_Code_of_Practice_revision_2013_-_final.pdf. Accessed 13 Oct 2016.

[19] Pebody R, Warburton F, Ellis J, Andrews N, Potts A, Cottrell S, et al. Effectiveness of seasonal influenza vaccine for adults and children in preventing laboratory-confirmed influenza in primary care in the United Kingdom: 2015/16 end-of-season results. Euro Surveill. 2016;21. doi:10.2807/1560-7917.ES.2016.21.38.30348 10.2807/1560-7917.ES.2016.21.38.30348 PMC5073201 27684603

[20] Eames KT, Tilston NL, Brooks-Pollock E, Edmunds WJ (2012). Measured dynamic social contact patterns explain the spread of H1N1v influenza. PLoS Comput Biol..

[21] Belongia EA, Kieke BA, Donahue JG, Greenlee RT, Balish A, Foust A (2009). Effectiveness of inactivated influenza vaccines varied substantially with antigenic match from the 2004–2005 season to the 2006–2007 season. J Infect Dis..

[22] Nordin J, Mullooly J, Poblete S, Strikas R, Petrucci R, Wei F (2001). Influenza vaccine effectiveness in preventing hospitalizations and deaths in persons 65 years or older in Minnesota, New York, and Oregon: data from 3 health plans. J Infect Dis..

[23] Van Bellinghen L-A, Meier G, Van Vlaenderen I, Pitman R, Melegaro A, Gelb D (2014). The potential cost-effectiveness of quadrivalent versus trivalent influenza vaccine in elderly people and clinical risk groups in the UK: a lifetime multi-cohort model. PLoS ONE..

[24] Hurwitz ES, Haber M, Chang A, Shope T, Teo ST, Giesick JS (2000). Studies of the 1996–1997 inactivated influenza vaccine among children attending day care: immunologic response, protection against infection, and clinical effectiveness. J Infect Dis..

[25] NICE. Guide to the methods of technology appraisal 2013. 2013. https://www.nice.org.uk/article/pmg9/chapter/foreword. Accessed 13 Oct 2016.

[26] de Boer P, van Maanen B, Damm O, Ultsch B, Dolk FC, Crépey P (2017). A systematic review of the health economic consequences of quadrivalent influenza vaccination. Expert Rev Pharmacoecon Outcomes Res.

[27] Langley JM, Wang L, Aggarwal N, Bueso A, Chandrasekaran V, Cousin L (2015). Immunogenicity and reactogenicity of an inactivated quadrivalent influenza vaccine administered intramuscularly to children 6 to 35 months of age in 2012-2013: a randomized, double-blind, controlled, multicenter, multicountry, clinical trial. J Pediatr Infect Dis Soc..

[28] Jain VK, Rivera L, Zaman K, Espos RA, Sirivichayakul C, Quiambao BP (2013). Vaccine for prevention of mild and moderate-to-severe influenza in children. N Engl J Med..

