# Supplementary material for: Cost-effectiveness analysis of quadrivalent seasonal influenza vaccines in England
Source: BMC Med. 2017 Sep 8;15:166. doi: 10.1186/s12916-017-0932-3 (PMC5590113; doi:10.1186/s12916-017-0932-3)
Supplement: Additional file 1: — Assessing the impact of different paediatric programmes, sensitivity analyses for programmes 2 and 3, and cost-effectiveness acceptability curves. (DOCX 44 kb) [file 12916_2017_932_MOESM1_ESM.docx]

# Additional file 1

## Assessing the impact of different paediatric programmes on the cost-effectiveness of QIIV programmes for adults

| Cost per QALY | Paediatric programe | High risk 18-64 years | HR 18-64 + Elderly 65+ |
| --- | --- | --- | --- |
| £20,000 | QLAIV & QIIV | £1.84 | £0.20 |
| £20,000 | LAIV & TIIV | £3.55 | £0.29 |
| £20,000 | No paediatric programme | £3.76 | £0.41 |

***Table S1****: The maximum incremental vaccine cost of the quadrivalent vaccines for high risk individuals aged 18-64 years and elderly individuals aged 65+ years for three different paediatric vaccination programmes.*

The estimated maximum incremental cost per dose of quadrivalent vaccines over trivalent vaccines for the high risk and elderly cohorts when no paediatric vaccination programme is implemented is likely to be higher than the £3.76 and £0.41 stated, as these estimates do not account for the change in the burden of influenza A attributable to the paediatric vaccination programme.

## Sensitivity analyses for other programmes

### Programme 2


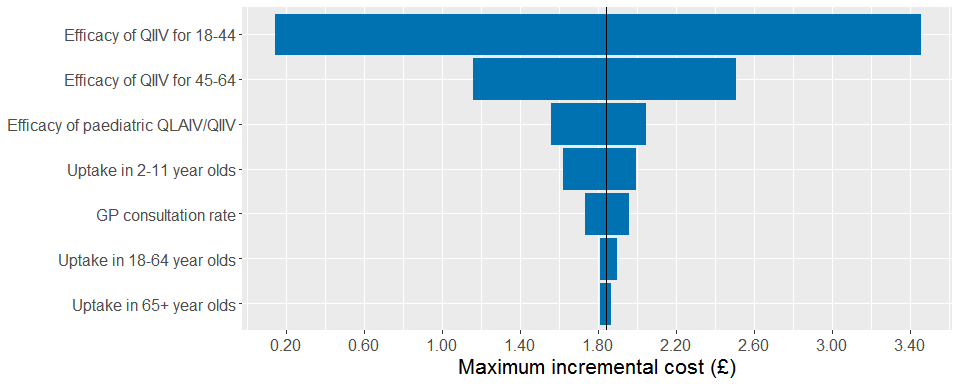


***Figure S1****: Tornado plot showing the estimated change in the maximum incremental cost-per-dose of the quadrivalent vaccines for programme 2, assuming a WTP threshold of £20,000 per QALY.*

### Programme 3


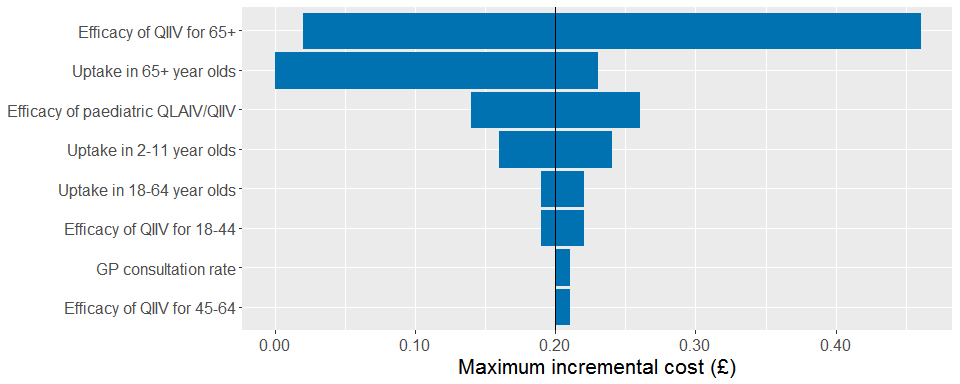


***Figure S2****: Tornado plot showing the estimated change in the maximum incremental cost-per-dose of the quadrivalent vaccines for programme 3, assuming a WTP threshold of £20,000 per QALY.*

## Cost-effectiveness acceptability curves


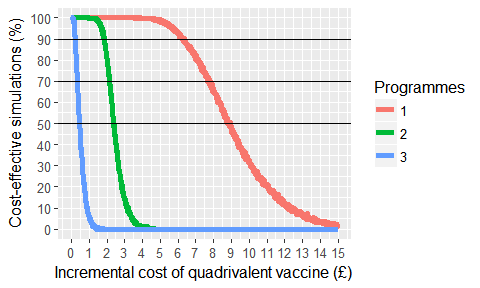


***Figure S3****: Cost-effectiveness analysis curves to show the percentage of simulations for each vaccination programme that would be cost-effective to a threshold of £20,000.*

| Cost per QALY | Programme 1 | Programme 2 | Programme 3 |
| --- | --- | --- | --- |
| **90% of simulations cost-effective** |  |  |  |
| £10,000 | £3.69 | £1.03 | £0.11 |
| £15,000 | £5.04 | £1.44 | £0.16 |
| £20,000 | £6.36 | £1.84 | £0.20 |
| £25,000 | £7.58 | £2.25 | £0.26 |
| £30,000 | £8.89 | £2.66 | £0.31 |
| **70% of simulations cost-effective** |  |  |  |
| £10,000 | £4.51 | £1.18 | £0.17 |
| £15,000 | £6.21 | £1.66 | £0.25 |
| £20,000 | £7.77 | £2.14 | £0.33 |
| £25,000 | £9.41 | £2.62 | £0.42 |
| £30,000 | £11.01 | £3.11 | £0.49 |
| **50% of simulations cost-effective** |  |  |  |
| £10,000 | £5.11 | £1.31 | £0.23 |
| £15,000 | £7.00 | £1.83 | £0.34 |
| £20,000 | £8.92 | £2.38 | £0.45 |
| £25,000 | £10.78 | £2.92 | £0.56 |
| £30,000 | £12.61 | £3.45 | £0.67 |

***Table S2****: The maximum incremental vaccine cost of the quadrivalent vaccines for five different WTP thresholds and three different criteria on the proportion of simulations above the WTP threshold`.*
